# Directional Spatial and Spectral Attention Network (DSSA Net) for EEG-based emotion recognition

**DOI:** 10.3389/fnbot.2024.1481746

**Published:** 2025-01-07

**Authors:** Jiyao Liu, Lang He, Haifeng Chen, Dongmei Jiang

**Affiliations:** ^1^School of Computer Science, Northwestern Polytechnical University, Xi'an, China; ^2^School of Computer Science and Technology, Xi'an University of Posts and Telecommunications, Xi'an, Shaanxi, China; ^3^School of Electronic Information and Artificial Intelligence, Shaanxi University of Science and Technology, Xi'an, China

**Keywords:** EEG, emotion recognition, spectral attention, position attention, temporal attention, directional spatial attention

## Abstract

Significant strides have been made in emotion recognition from Electroencephalography (EEG) signals. However, effectively modeling the diverse spatial, spectral, and temporal features of multi-channel brain signals remains a challenge. This paper proposes a novel framework, the Directional Spatial and Spectral Attention Network (DSSA Net), which enhances emotion recognition accuracy by capturing critical spatial-spectral-temporal features from EEG signals. The framework consists of three modules: Positional Attention (PA), Spectral Attention (SA), and Temporal Attention (TA). The PA module includes Vertical Attention (VA) and Horizontal Attention (HA) branches, designed to detect active brain regions from different orientations. Experimental results on three benchmark EEG datasets demonstrate that DSSA Net outperforms most competitive methods. On the SEED and SEED-IV datasets, it achieves accuracies of 96.61% and 85.07% for subject-dependent emotion recognition, respectively, and 87.03% and 75.86% for subject-independent recognition. On the DEAP dataset, it attains accuracies of 94.97% for valence and 94.73% for arousal. These results showcase the framework's ability to leverage both spatial and spectral differences across brain hemispheres and regions, enhancing classification accuracy for emotion recognition.

## 1 Introduction

Emotion plays a crucial role in our daily lives, manifesting through various forms such as auditory cues, facial expressions, and physiological signals (He L. et al., 2022). Among these, electroencephalogram (EEG) signals are particularly noteworthy due to their objective nature and resistance to falsification, making them a reliable indicator of different emotional states. With the rapid advancements in EEG acquisition technology and the evolution of machine learning techniques, the field of EEG-based emotion recognition has gained significant attention and made remarkable progress (Wang et al., 2024).

EEG exhibits distinct features across temporal, spatial, and frequency domains in different emotions (Li et al., 2019; Mognon et al., 2011). For example, during states of happiness or sadness, EEG may show different wave amplitudes and frequency distributions (Alarcao and Fonseca, 2017). Specific EEG frequency bands such as alpha waves (8–12 Hz) and beta waves (12–30 Hz) are known to be associated with different emotional states. Alpha waves are generally linked to relaxation and calmness (Davidson et al., 2000), while beta waves often indicate alertness and tension (Ray and Cole, 1985). Beyond analyzing these features individually, recent studies have demonstrated that the fusion of spectral and spatial features can significantly enhance emotion recognition performance. For example, a spatio-temporal self-constructing graph neural network (ST-SCGNN) effectively combines spectral information from EEG frequency bands with spatial activation patterns across different brain regions, leveraging their complementary strengths for cross-subject emotion recognition (Pan et al., 2023). This combination allows for a more comprehensive understanding of how different frequency bands manifest across specific spatial areas of the brain, providing a richer representation of emotional states. In addition to frequency and spatial attributes, temporal features are crucial for capturing the dynamic changes in brain activity that occur over time. For example, temporal models like Temporal Convolutional Networks (TCN) have been used to encode these time-dependent characteristics, significantly improving the performance of EEG-based cross-domain emotion recognition (He Z. et al., 2022). This highlights the importance of temporal modeling for capturing the evolving nature of emotional responses in EEG signals.

Neuroscientific research suggests that emotional processing involves a closely integrated system across both hemispheres and the anterior-posterior regions of the brain, with significant overlap in their roles. The left hemisphere tends to be more involved in processing emotions related to approach behaviors and positive affect, such as happiness and satisfaction, whereas the right hemisphere may play a greater role in processing emotions related to withdrawal behaviors and negative affect, such as sadness and fear (Celeghin et al., 2017). Similarly, the anterior brain regions, particularly the frontal lobe, are associated with heightened activity in response to positive emotions, while posterior regions, including the occipital lobe, show increased activity when processing visual emotional stimuli (Kringelbach and Berridge, 2010; Abdel-Ghaffar et al., 2024).

In addition to these spatial dimensions, specific frequency bands are also tied to certain brain regions and emotional states. For instance, alpha waves (8–12 Hz) are typically dominant in the posterior brain regions, particularly in occipital and parietal areas, and are associated with relaxation and calmness. In contrast, beta waves (12–30 Hz) are more prominent in the frontal regions, reflecting alertness and cognitive processing. These frequency bands exhibit different strengths depending on whether the signals come from the anterior-posterior or left-right axes, which reflect emotional lateralization (Davidson et al., 2000).

This connection between horizontal and vertical orientations and spectral characteristics is supported by the psychological understanding that the anterior-posterior axis is more involved in processing emotional stimuli, while the left-right axis helps differentiate positive and negative emotions (Davidson, 1992). Our proposed model leverages these insights, enabling the attention mechanism to focus on specific frequency bands according to the brain regions, improving emotion recognition performance.

Emotion classification based on EEG signals has made significant strides in recent years, reflecting the objective and precise emotional states of humans through electrophysiological manifestations. Wang et al. (2011) utilized a support vector machine (SVM) classifier to differentiate various emotional states, but their method struggled with the complex and high-dimensional nature of EEG data. Alhagry et al. (2017) employed a two-layer Long Short-Term Memory (LSTM) network, which captured temporal dependencies in the EEG signals and achieved satisfactory results. Despite this progress, there remains a need for the comprehensive fusion of features from spatial, temporal, and frequency domains. Tao et al. (2020) introduced an attention-based convolutional recurrent neural network (ACRNN) that employs a channel-wise attention mechanism with a CNN for adaptive spatial feature extraction and extended self-attention within an RNN to analyze temporal dynamics in EEG signals, considering crucial information across spatial and temporal domains. To further take advantage of spectral information, Xiao et al. (2022) utilized a CNN with spectral and spatial attention mechanisms to dynamically adjust weights across different brain regions and frequency bands, and incorporatedd a temporal attention mechanism within a bidirectional LSTM to analyze temporal dependencies in the data. Zhu et al. (2024) developed the Self-Organized Graph Pseudo-3D Convolution (SOGPCN). It uses a self-organizing graph convolution module to extract the spatial features from each frequency band and 3D-CNN layers followed by dot product attention layers to focus on valuable information, and utilizes an LSTM layer to model the temporal dynamics. In our previous work (Liu et al., 2021), we developed a 3D convolutional network (3D-CNN) that integrates a parallel positional-spectral-temporal attention module to learn crucial information across different domains for EEG emotion recognition. Although the aforementioned studies have effectively incorporated spatial, temporal, and spectral information, they typically viewed the EEG's spatial domain as a unified whole while ignoring the functional differences between anterior-posterior and left-right brain regions.

In this paper, we propose a Directional Spatial and Spectral Attention Network (DSSA Net) that enhances spatial, spectral, and temporal features, specifically emphasizing the attention across complementary orientations in the brain area. It is composed of the Position Attention (PA), Spectral Attention (SA), and Temporal Attention (TA) modules. The PA Module, consisting of the vertical attention (VA) branch and horizontal attention (HA) branch, learns the spatial attentions on orthogonal orientations from both anterior-posterior and left-right brain regions, While the SA Module learns the attention on different frequency bands. Unlike traditional undirected models, which treat the spatial dimensions of EEG signals uniformly, DSSA Net leverages the directional nature of brain activities by separately modeling the attention across anterior-posterior and left-right brain regions. This directional approach aligns with established neuroscientific findings that different hemispheres and brain regions are differentially involved in emotional processing, allowing the network to more accurately capture emotion-specific patterns in EEG data. These two modules produce a 3D attention map that enhances important frequency bands within the spatial context. Subsequently, the Temporal Attention (TA) Module models the temporal dynamics of the enhanced feature sequence using a Transformer encoder. In this way, our proposed framework captures the critical features across the spatial, spectral, and temporal domains, highlighting the contributions of various brain regions in the anterior-posterior and left-right directions on emotion recognition. The primary contributions of this paper are as follows:

- In this paper, we propose the Directional Spatial and Spectral Attention Network (DSSA Net), a novel EEG emotion recognition framework, which emphasizes the contributions of various brain regions on emotion recognition, while specially taking into account the functional differences between the anterior and posterior as well as the left and right brain regions, and the specific frequency bands associated with different emotional states.
- We conducted comprehensive experiments on benchmark datasets such as *DEAP, SEED*, and *SEED-IV*, which confirmed that the DSSA Net significantly outperforms most existing methods in emotion recognition tasks. This superior performance is largely due to its effective analysis of EEG signals from distinct brain orientations, notably the anterior-posterior and left-right directions. Ablation studies further confirm the critical importance of cohesively analyzing spatial directions and spectral features.

## 2 Related work

Emotion classification based on EEG signals is a meaningful research direction. As the electrophysiological manifestation of the central nervous system, EEG objectively and precisely reflects the real emotional states of humans. With the development of artificial intelligence technology, emotion recognition has become a hot research topic in human-computer interaction.

### 2.1 Traditional machine learning based methods

In recent years, several studies have applied traditional machine learning techniques to EEG-based emotion recognition. Koelstra et al. (2011), Wang et al. (2011), and Bahari and Janghorbani (2013) have employed Gaussian Naive Bayes, Support Vector Machines (SVM), and a combination of recurrence plot analysis with k-nearest neighbor classifiers, respectively, to analyze various emotional states. Additionally, Jiang et al. (2019) and Zhang et al. (2024) have enhanced cross-subject emotion recognition by integrating decision tree classifiers and a dynamically optimized Random Forest model, respectively, each enhanced by algorithmic adaptations for better performance. Xu X. et al. (2024) addressed the challenges of small EEG sample sizes and high feature dimensionality by leveraging both local and global label relevance for feature selection. It employs orthogonal regression to map EEG features into a low-dimensional space to capture local label correlations, and then integrates global label correlations from the original multi-dimension emotional label space. Xu et al. (2021) optimized EEG emotion recognition by globally evaluating and reducing feature redundancy, selecting informative and non-redundant features to improve performance across various datasets.

### 2.2 Deep learning based methods

Compared with traditional methods, deep learning technologies offer significant advantages in high-level representation and end-to-end training schemes. Al-Nafjan et al. (2017) utilized Deep Neural Networks (DNN) with Power Spectral Density (PSD) features to identify human emotions. Further considering the temporal characteristics of EEG, Alhagry et al. (2017) achieved satisfactory emotion recognition results by employing a two-layer Long Short-Term Memory (LSTM) network using EEG signals as input. Hefron et al. (2017) implemented LSTM-based Recurrent Neural Networks (RNNs) to model the time dependence of cognitive-related EEG signals. However, these studies did not fully exploit the multi-dimensional information available in EEG signals. To address the limitations of these approaches, some researchers have explored multi-dimensional features in EEG-based emotion recognition. Bashivan et al. (2015) proposed a deep recursive convolutional neural network (R-CNN) for EEG-based cognitive and mental load classification tasks, incorporating spatial and temporal dimensions. Zhang et al. (2017) developed a deep CNN model to learn robust spatio-temporal feature representations of raw EEG data for motion intention classification. Despite considering multi-dimensional features, these studies still have limitations. They often overlook critical aspects such as the frequency domain or the dynamic changes in EEG signals over longer periods, which are essential for a more comprehensive understanding of emotional states. Wu et al. (2024) enhanced the adaptability of EEG emotion recognition by using a dual-graph structure to capture emotion-relevant and emotion-irrelevant features, and applying orthogonal purification to reduce redundancy and align feature spaces across subjects.

### 2.3 Attention based methods

To further enhance the analysis, attention mechanisms have been introduced to effectively capture and emphasize the most relevant features across different dimensions, providing a more detailed and accurate understanding of EEG-based emotion recognition. Building on this foundation, several advanced approaches have emerged. Tao et al. (2020) proposed an attention-based convolutional recurrent neural network (ACRNN) to extract more discriminative features and improve emotion recognition accuracy. The model employed a channel-wise attention mechanism and a CNN to adaptively extract spatial information, which further integrated extended self-attention with an RNN to explore temporal information based on intrinsic similarities within the EEG signals. To further take advantage of the different importance of frequency band features, Xiao et al. (2022) introduced the four-dimensional attention-based neural network (4D-aNN) for EEG emotion recognition. The 4D-aNN employs spectral and spatial attention mechanisms to dynamically adjust the weights assigned to different brain regions and frequency bands. A convolutional neural network (CNN) processes this spectral and spatial information. Additionally, the model integrates a temporal attention mechanism within a bidirectional Long Short-Term Memory (LSTM) network, enabling the exploration of temporal dependencies in the 4D EEG representations, aiming to enhance the utilization of comprehensive signal information for emotion recognition. Zhang et al. (2023) introduced an attention-based hybrid deep learning model for EEG emotion recognition. The model starts by extracting differential entropy features from EEG data, organized by electrode positions. It uses a convolutional encoder to capture spatial features and a band attention mechanism to weight different frequency bands. A long short-term memory (LSTM) network with a time attention mechanism then extracts and highlights key temporal features, enhancing classification accuracy. To further consider spatial correlations and temporal context information, Zhu et al. (2024) presented the Self-Organized Graph Pseudo-3D Convolution (SOGPCN). Unlike traditional methods that construct static graph structures for brain channels, SOGPCN dynamically addresses the varying spatial relationships between electrodes across different frequency bands. The process begins with creating a self-organizing map for each channel within each frequency band, identifying the 10 most relevant channels. Graph convolution then captures spatial relationships within these maps. Subsequently, pseudo-three-dimensional convolution paired with dot product attention is used to extract temporal features from the EEG sequences. Finally, an LSTM layer is utilized to learn contextual information between adjacent time-series data, aiming to improve the accuracy and reliability of emotion recognition from EEG signals.

Building on the significant advancements made in EEG-based emotion recognition through various attention-based models, it is evident that the incorporation of attention mechanisms in different dimensions significantly improves the feature extraction process. However, many existing methods still fail to fully integrate attention mechanisms across all dimensions, potentially missing crucial features. Additionally, even when these dimensions are considered, existing methods often fail to adequately explore the interactions between specific frequency bands and directional spatial positions within critical brain regions such as the left and right hemispheres and the anterior and posterior areas. These areas are essential for a nuanced understanding of emotional processing, yet they are frequently overlooked in the simultaneous analysis of spatial and spectral data. To address these issues, we introduce the DSSA Net, a framework that not only incorporates attention mechanisms across spatial, spectral, and temporal dimensions but also intricately links specific frequency bands to spatially distinct brain regions. This approach utilizes Positional Attention (PA) to highlight key positions related to emotion processing, which are directly associated with distinct frequency bands through the Spectral Attention (SA). By aligning spatial distinctions with spectral characteristics, our model enhances the detection of emotional states. Temporal Attention (TA) further prioritizes significant temporal slices, collectively enhancing the accuracy of emotion recognition.

## 3 Methodology

### 3.1 Overview of model structure

**Figure 1:**
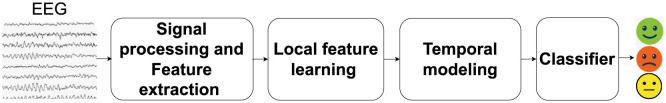
Overview of the DSSA Net framework for EEG-based emotion recognition. The framework includes: (1) Signal Processing and Feature Extraction, which segments EEG signals and extracts differential entropy features across five frequency bands; (2) Local Feature Learning, featuring Position Attention (PA) with Vertical (VA) and Horizontal (HA) branches for spatial focus, and Spectral Attention (SA) for frequency-specific information; (3) Temporal Modeling (TA) using a Transformer encoder to capture temporal dynamics, followed by an MLP head for emotion prediction. The 3D attention map combines spatial and spectral insights to enhance feature representation.

The proposed DSSA Net framework comprising three modules is illustrated in Figure 1. The signal processing and feature extraction module segments the EEG signals into non-overlapping samples. For each sample, it extracts the differential entropy features across five frequency bands, and reorganizes them into a 3D feature representation following the layout of EEG electrodes.

The design of the Position Attention (PA) module, which includes vertical attention (VA) and horizontal attention (HA), is grounded in neuroscientific evidence that different brain regions play specific roles in emotional processing. The VA focuses on anterior-posterior brain activity, capturing the differences between frontal and occipital regions, which are known to be involved in cognitive and emotional responses. The HA focuses on left-right differences, reflecting the lateralization of emotions, where the left hemisphere is more associated with positive emotions and the right hemisphere with negative emotions (Davidson, 1992). These attentions allow the model to align with the spatial structure of brain activity as outlined in the introduction.

The local feature learning module contains a Position Attention (PA) branch with vertical attention (VA) and horizontal attention (HA) to emphasize the significant brain regions by analyzing comprehensive directional activation patterns, and a Spectral Attention (SA) branch to identify the frequency bands that are essential for emotion recognition.

The choice of the Spectral Attention (SA) branch is aimed at leveraging the frequency-specific emotional information, such as alpha and beta waves, which are linked to relaxation and alertness, respectively. This complements the spatial focus of VA and HA by allowing the model to focus on the most informative frequency bands.

The 3D attention map, learned from the VA, HA, and SA branches, is then applied to enhance the 3D feature representation via element-wise multiplication. The Temporal Modeling (TA) module leverages a Transformer encoder to capture the temporal dynamics among the enhanced feature representation sequence of the EEG signals. Finally, the attention-weighted EEG features are averaged along the temporal domain and fed into a multilayer perceptron (MLP) head to predict the emotional state.

### 3.2 Signal processing and feature organization

In this section, we introduce the form of EEG signal processing and feature representation. Firstly, we partition the EEG signals into samples of *T* seconds, which are assigned the same emotion labels as those of their original EEG signals. For each sample, the EEG signal of each channel is first passed through a Butterworth bandpass filter between 0.5 and 50 Hz, then sliced into 1-s non-overlapping segments. For each segment, differential entropy (DE) features are extracted at five frequency bands: δ (1–3 Hz), θ (4–7 Hz), α (8–13 Hz), β (14–30 Hz), and γ (31–50 Hz), resulting in a five-dimensional feature vector.

Therefore, for the total *C* channels of the *t*-th second of a sample, a 2D feature matrix $f_{t}\in {\mathbb{R}}^{C\times S}$ is obtained, where *S* = 5 represents the number of spectral bands.

The extracted DE features in each band correspond to different physiological characteristics of brain activity. For example, α waves (8–13 Hz) are dominant in the posterior regions and are associated with states of relaxation and calmness, reflecting reduced cognitive load and increased internal focus. β waves (14–30 Hz), on the other hand, are prominent in the frontal regions during states of alertness, focus, and even stress, indicating active cognitive processing. δ waves (1–3 Hz) are often linked to deep sleep and restorative brain activities, which can affect the baseline emotional state. θ waves (4–7 Hz) play a role in memory and emotional regulation, and often become more pronounced during meditative states or drowsiness. γ waves (31–50 Hz) are associated with high-frequency cognitive processing and attention, particularly during intense emotional experiences. These band-specific characteristics align with the brain's horizontal and vertical differentiation in emotional processing, as described in the introduction. By reorganizing the EEG data into a 3D feature representation following the electrode layout, our model integrates both the spatial and spectral information in a manner that reflects these known neural patterns.

**Figure 2:**
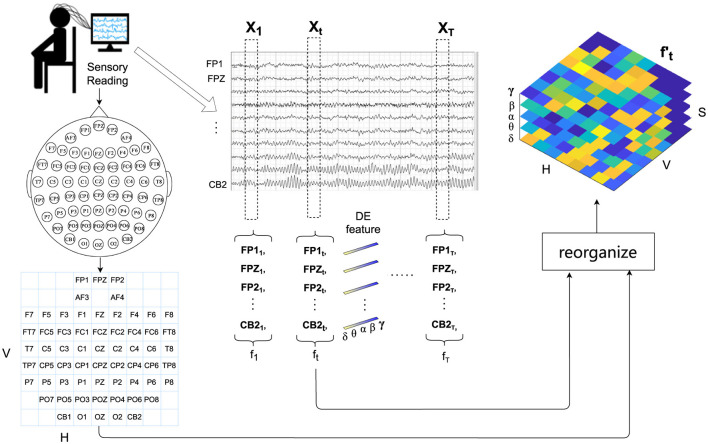
Detailed workflow of signal processing and feature organization for EEG-based emotion recognition. This includes filtering, segmentation, differential entropy feature extraction across five frequency bands, and the reorganization of extracted features into a 3D representation following the EEG electrode layout for enhanced spatial and spectral analysis.

To fully utilize the positional information of EEG electrodes, following the *H* × *V* grid layout of EEG electrodes as shown on the left of Figure 2, we reorganize $f_{t}\in {\mathbb{R}}^{C\times S}$ of *C* EEG channels into a reshaped EEG feature representation $f_{t}^{\prime }\in {\mathbb{R}}^{H\times V\times S}$. For the empty grids in the layout that do not match the actual electrodes, considering that the electrical activity of the brain is spatially continuous and signals from adjacent electrodes are usually highly correlated (Valk et al., 2020), we use bi-linear interpolation to fill in the missing values.

### 3.3 Local feature learning

In psychological and cognitive research, studies have consistently demonstrated differential activation patterns between left and right brain hemispheres during various cognitive tasks and emotional processing. For instance, the lateralization theory suggests that emotions may be processed differently in the left and right hemispheres of the brain, leading to distinct activation patterns (Davidson, 1992). Additionally, investigations into the anterior-posterior brain regions have highlighted the significance of anterior and posterior brain areas in emotional processing (Harmon-Jones and Gable, 2018). These studies indicate that different brain regions along both horizontal (left-right) and vertical (anterior-posterior) dimensions play crucial roles in emotional processing. Given the importance of these different brain regions in emotional processing, there is a growing need to explore attention mechanisms that can capture both the anterior-posterior and left-right directional cues in neural data processing. Furthermore, in the realm of spectral analysis, research has underscored the importance of different frequency bands in encoding emotional states. Studies have shown that specific frequency bands, such as alpha, beta, and gamma, exhibit distinct patterns of activity corresponding to different emotional experiences (Jenke et al., 2014). Hence, capturing effective spectral information is paramount in accurate decoding of emotional states from neural signals.

**Figure 3:**
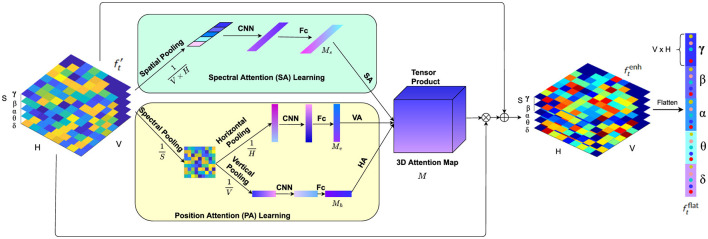
The local feature learning framework consisting of the PA module and SA module. While the PA module (faint yellow) highlights more critical features among left and right brain regions as well as protocerebrum and tritocerebrum, the SA module (faint green) gives prominence to the key EEG spectral bands for emotion recognition.

Motivated by these findings, we propose a novel local feature learning framework as depicted in Figure 3, which consists of two pivotal modules: the Position Attention (PA) module and the Spectral Attention (SA) module. The PA module captures vital cues from both the anterior-posterior and left-right brain regions, while the SA module emphasizes crucial spectral features relevant to emotional states. The outputs of these modules compose a 3D attention map, which is used to weight the EEG feature representation by element-wise product. This combined attention mechanism highlights more important spectral bands among the spatial regions in the anterior-posterior and left-right directions. The weighted feature representation is then integrated with the original feature to obtain a comprehensive representation encapsulating salient spatial and spectral information, which is then flattened into a feature vector following the grid layout within each frequency band.

#### 3.3.1 Position attention module

Studies have highlighted the significance of both anterior-posterior (anterior and posterior) and left-right (hemispheric) brain regions in emotional processing (Harmon-Jones and Gable, 2018). Furthermore, research in neuroscience has consistently demonstrated that different brain regions activate differently during various emotional tasks. This phenomenon, known as lateralization, indicates that the left and right hemispheres of the brain exhibit distinct activation patterns when processing emotions (Davidson, 1992). These differential activation patterns suggest that capturing spatial attention across these dimensions is crucial for understanding and classifying emotions. Inspired by these insights, we propose a Position Attention (PA) module that consists of two branches to learn spatial attention masks in both vertical and horizontal directions to highlight valuable brain regions for emotion classification: the vertical attention (VA) branch and the horizontal attention (HA) branch. The VA branch captures the differential activation and highlights more important features among the anterior-posterior brain regions, while the HA module highlights the salient information on the left-right hemispheric regions. Unlike previous spatial attention methods (Tao et al., 2020), which employ channel-wise attention to enhance feature representations by assigning weights to different electrodes independently, our method considers the significance of positions among the left-right and anterior-posterior brain regions by using both horizontal and vertical attention mechanisms. This approach enables the model to simultaneously capture spatial dependencies in both directions, enhancing the ability to detect complex interactions between different brain regions and improving the accuracy of emotion classification.

Specifically, the PA module operates as follows. Given the input EEG feature representation $f_{t}^{\prime }$, firstly the differential entropy (DE) features of all the *S* frequency bands on each grid are pooled, obtaining the average spectral distribution on the grid layout of the EEG electrodes. Then the horizontal pooling is performed to get the average spectral feature $R_{v}\left(f_{t}^{\prime }\right)$ along the anterior-posterior brain region direction, and vertical pooling is performed to obtain the average spectral feature $R_{h}\left(f_{t}^{\prime }\right)$ along the left-right hemispheric region direction.


(1) $$\begin{array}{l} R_{v}\left(f_{t}^{\prime }\right)=\frac{1}{S\times H}\overset{S}{\underset{i=1}{\sum }}\overset{H}{\underset{j=1}{\sum }}f_{t_{ij}}^{\prime } \end{array}$$

(2) $$\begin{array}{l} R_{h}\left(f_{t}^{\prime }\right)=\frac{1}{S\times V}\overset{S}{\underset{i=1}{\sum }}\overset{V}{\underset{k=1}{\sum }}f_{t_{ik}}^{\prime } \end{array}$$

where *i*, *j* and *k* represents the indices for the spectral, horizontal and vertical dimensions of the input tensor, respectively.

While pooling along these directions effectively summarizes spectral features, it can result in the loss of certain fine-grained spatial details. To address this, the attention mechanisms in the orthogonal directions (VA and HA branches) help to retain important spatial relationships that might be compressed during the pooling process. Additionally, the original feature maps, which contain the full spatial distribution, are reintroduced in later stages, ensuring that the overall spatial structure is preserved.

Next, fully connected layers and activation functions are applied to generate the attention masks:


(3) $$\begin{array}{l} M_{v}=\phi \left(ReLU\left(f_{c}\left(f_{1d}\left(R_{v}\left(f_{t}^{\prime }\right)\right)\right)\right)\right) \end{array}$$

(4) $$\begin{array}{l} M_{h}=\phi \left(ReLU\left(f_{c}\left(f_{1d}\left(R_{h}\left(f_{t}^{\prime }\right)\right)\right)\right)\right) \end{array}$$

where *f*_1*d*_ represents the 1D convolution operation. *f*_*c*_ denotes the fully connected layer, which is used to combine the extracted features into a more compact and representative form. To ensure that the dimensionality of the EEG features remains consistent throughout the convolutional layers, we applied padding settings that preserve the dimensions between input and output after each convolution operation. This approach is critical for maintaining the alignment between the attention masks and the feature maps, as it ensures that the recalibrated features from the attention modules can be directly applied to the corresponding spatial and spectral regions without any spatial distortion or resizing. By maintaining consistent dimensions, the model can effectively focus on relevant regions, leading to better emotion recognition performance. This strategy also prevents the need for additional resizing steps, which could introduce artifacts or reduce the effectiveness of the attention mechanisms. By preserving the spatial resolution of the feature maps, we ensure that the attention masks are directly applied without requiring additional adjustments. *ReLU* denotes the ReLU activation function, which is applied to the hidden layers to introduce non-linearity. This allows the model to capture complex patterns and relationships within the data by enabling the learning of non-linear feature transformations. The use of ReLU helps prevent issues such as the vanishing gradient problem and promotes efficient gradient-based optimization.

ϕ stands for the Softmax function, which is applied at the output of the fully connected layer. Softmax normalizes the outputs into a probability distribution, where the sum of the outputs equals 1, making it suitable for tasks where the output needs to represent mutually exclusive probabilities. This ensures that each output neuron represents the probability of a particular class or state, which is critical for the classification task in our model. By combining ReLU in the hidden layers and Softmax at the output, the model is able to first learn non-linear transformations of the input data, and then produce interpretable probabilistic outputs that can be used for classification. This combination of non-linearity from ReLU and the probabilistic nature of Softmax is essential for learning complex patterns while maintaining an interpretable output space.

Through the position attention learning module, the learned *V* dimension vector *M*_*v*_ and *H* dimension vector *M*_*h*_ can be used as the attention masks to highlight the brain regions in the vertical and horizontal directions, respectively.

#### 3.3.2 Spectral attention module

The spectral analysis of EEG signals has shown that different frequency bands play crucial roles in encoding emotional states. For instance, the delta (1–3 Hz) band is often associated with deep sleep and unconscious processes, the theta (4–7 Hz) band with meditation and memory retrieval, the alpha (8–13 Hz) band with relaxation and mental coordination, the beta (14–30 Hz) band with active thinking and focus, and the gamma (31–50 Hz) band with higher cognitive functions and information processing (Jenke et al., 2014). These distinct patterns of activity in different frequency bands suggest that capturing spectral attention across these bands is essential for accurate emotion classification. Based on these findings, we propose a Spectral Attention (SA) module to learn a spectrum attention mask that emphasizes on more significant frequency bands for emotion classification. Unlike traditional statistical methods or Principal Component Analysis (PCA), which rely on fixed linear transformations to reduce dimensionality, the SA module highlights key spectral features by focusing on the differential energy spectrum of these bands when the brain is subjected to various emotional stimuli. This allows the model to capture discriminative EEG frequencies, thereby enhancing the performance of the emotion classifier.

Specifically, given the EEG feature representation $f_{t}^{\prime }$, the SA module operates as follows. First, the position pooling is performed to summarize the spectral features on all the grids of the layout:


(5) $$\begin{array}{l} R_{s}\left(f_{t}^{\prime }\right)=\frac{1}{V\times H}\overset{V}{\underset{k=1}{\sum }}\overset{H}{\underset{j=1}{\sum }}f_{t_{kj}}^{\prime } \end{array}$$

Next, the attention weight vector is computed:


(6) $$\begin{array}{l} M_{s}=\phi \left(\text{ReLU}\left(\text{fc}\left(f_{1d}\left(R_{s}\left(f_{t}^{\prime }\right)\right)\right)\right)\right) \end{array}$$

where *f*_1*d*_ represents the 1D convolution operation, fc denotes the fully connected layer, and ReLU denotes the ReLU activation function and ϕ stands for softmax function. This process generates a *S* dimension vector as the attention mask of the spectral features.

#### 3.3.3 3D attention map weighted local feature representation

After the attention masks (*M*_*v*_, *M*_*h*_, and *M*_*s*_) are learned, they are composed into a 3D attention map that integrates attention across horizontal (left-right), vertical (anterior-posterior), and spectral dimensions:


(7) $$\begin{array}{l} M=M_{v}⊗M_{h}⊗M_{s} \end{array}$$

where ⊗ represents the tensor product. The 3D attention map *M* effectively captures the salient spatial and spectral features by considering both horizontal and vertical orientations of the brain, as well as the most relevant frequency bands. This comprehensive map ensures that the recalibrated features are enriched with region-specific and frequency-specific information.

The attention map is then used to recalibrate the local feature representation as:


(8) $$\begin{array}{l} f_{t}^{\text{rec}}=f_{t}^{\prime }\times M \end{array}$$

where × represents element-wise multiplication. In this step, $f_{t}^{\text{rec}}$ becomes an enhanced feature representation that integrates the spatial attention from the horizontal and vertical brain axes and spectral attention from frequency bands. This recalibration process highlights the most informative regions and frequencies in the EEG data, focusing on features that are most indicative of emotional states.

The recalibrated feature $f_{t}^{\text{rec}}$ is then combined with the original feature $f_{t}^{\prime }$ generating an enhanced comprehensive representation $f_{t}^{\text{enh}}$ of the EEG signals:


(9) $$\begin{array}{l} f_{t}^{\text{enh}}=f_{t}^{\prime }+f_{t}^{\text{rec}} \end{array}$$

The enhanced feature $f_{t}^{\text{enh}}$, encapsulating the salient spatial and spectral information, is then flattened into a *V* × *H* × *S* dimension feature vector $f_{t}^{\text{flat}}$ following the grid layout within each frequency band. For an EEG sample of *T* seconds, the flattened outputs of the local feature learning module for all the *T* segments are assembled into a feature sequence $F^{\prime }=\left\{f_{1}^{flat},f_{2}^{flat},...,f_{T}^{flat}\right\}$, which is then input into a Transformer encoder for temporal modeling.

### 3.4 Temporal modeling

**Figure 4:**
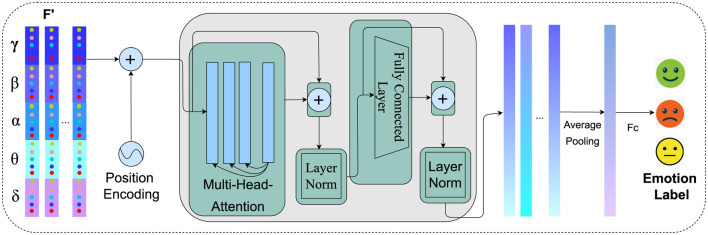
Temporal modeling of spatial-spectral EEG features using transformer encoder. The process integrates positional embeddings, multi-head attention, and feedforward networks to capture intricate temporal dynamics, resulting in enhanced feature representations for emotion recognition.

Studies have demonstrated that effectively learning the temporal dynamics from EEG is crucial for accurately interpreting neural responses (Tong et al., 2024; Ding and Chen, 2024). In our study, the feature vector $f_{t}^{flat}$ from the local feature module is fed into the Transformer encoder. Positional embeddings are combined with each sequence to create an vector *X*(*t*) = {*X*(1), *X*(2), …, *X*(*T*)}. Within the Transformer encoder, each enhanced vector passes through the following steps for each layer: First, multi-head attention allows the model to focus on discriminative information of the sequence. The output of this attention process is then added to the original enhanced vector (residual connection) and normalized (Add & Norm). This output is subsequently processed through a feedforward network, followed by another Add and Norm step, producing a series of hidden states {*h*_1_, *h*_2_, …, *h*_*T*_}. These states encapsulate the temporal and spatial-spectral relationships within the EEG data. The output of these states undergoes average pooling to combine them into a fixed-length vector, which is then fed into a multilayer perceptron (MLP) for final emotion classification. The whole process is illustrated in Figure 4.

Before feeding the feature sequences into the Transformer model, positional embeddings are added to encapsulate the temporal information within the sequence. These embeddings are computed as follows:


(10) $$\begin{array}{l} \begin{array}{c} \mathrm{PE}\left(t,2i\right)=\sin\left(\frac{t}{10000^{2i/d}}\right)\\ \mathrm{PE}\left(t,2i+1\right)=\cos\left(\frac{t}{10000^{2i/d}}\right) \end{array} \end{array}$$

Here, *t* indicates the position of the frame within the sequence, *i* denotes the index of the dimension, and *d* represents the total number of dimensions in the feature vector. This approach allows the Transformer to better discern the relationships between frames by infusing the sequence with positional context. Upon integrating positional embeddings, the abstract feature vector $f_{t}^{flat}$ from the local feature learning module for is combined with these embeddings, resulting in the enhanced feature vector *X*(*t*):


(11) $$\begin{array}{l} X\left(t\right)=f_{t}^{flat}+PE\left(t\right) \end{array}$$

This enriched sequence *X* = {*X*(1), *X*(2), ..., *X*(*T*)} is then processed by the Transformer to model the temporal dynamics of the EEG signals. In the Transformer's encoder, as proposed by Vaswani et al. (2017), each element of the sequence undergoes a transformation where the attention mechanism computes scores by performing a dot product between the query *Q*, key *K*, and value *V* matrices:


(12) $$\begin{array}{l} \text{Attention}\left(Q,K,V\right)=\mathrm{softmax}\left(\frac{QK^{T}}{\sqrt{d_{k}}}\right)V \end{array}$$

where *d*_*k*_ is the dimension of the *Q* and *K* matrices. This facilitates the generation of a weighted representation of the inputs based on their relevance. The outputs are then aggregated using a multi-head attention mechanism:


(13) $$\begin{array}{l} \text{MultiHead}\left(Q,K,V\right)=\text{Concat}\left(head_{1},head_{2},\ldots ,head_{h}\right)W^{O} \end{array}$$

where *h* represents the number of attention heads, and *W*^*O*^ is a projection matrix. This mechanism enables simultaneous focus on different segments of the input sequence, enhancing the model's ability to process diverse features. Afterward, each sequence, enhanced by multi-head attention, is normalized through layer normalization:


(14) $$\begin{array}{l} Y=\mathrm{LN}\left(X+\text{MultiHead}\left(Q,K,V\right)\right) \end{array}$$

A feedforward network with ReLU activation then processes this normalized output:


(15) $$\begin{array}{l} \mathrm{FFN}\left(Y\right)=\mathrm{ReLU}\left(YW_{1}+b_{1}\right)W_{2}+b_{2} \end{array}$$

where *W*_1_ and *W*_2_ are learnable weight matrices, and *b*_1_ and *b*_2_ are bias vectors, facilitating the transformation and non-linearity in the model's feedforward layers. The output from the feedforward network is subsequently added back to *Y* and normalized:


(16) $$\begin{array}{l} \mathrm{Norm}\left(Y,\text{FFN}\left(Y\right)\right)=\mathrm{LN}\left(Y+\mathrm{FFN}\left(Y\right)\right) \end{array}$$

This sequence of operations is repeated across *L* layers within the encoder, creating a series of hidden states {*h*_1_, *h*_2_, ..., *h*_*T*_} that encapsulate both the detailed features and temporal relationships of the input sequence. These states form a comprehensive feature representation, which is then averaged into a fixed-length vector and fed into a multilayer perceptron for final classification. Through this structured approach, the Transformer effectively leverages local spatial-spectral characteristics and temporal dynamics, enhancing the performance of EEG emotion recognition tasks.

## 4 Experimental analysis

### 4.1 Datasets and experimental setup

We validate our model on SEED (Zheng et al., 2017; Duan et al., 2013) SEED-IV (Zheng et al., 2018) and DEAP (Koelstra et al., 2011) datasets, respectively.

**SEED** contains three different categories of emotion, namely positive, negative, and neutral. 62-channel EEG data of 15 subjects were collected while they were watching emotional videos which were carefully selected to elicit desired target emotions. For each subject, three sessions were recorded within an interval of about 1 week, each session contains 15 trails of about 4 min. For the subject-dependent experiments, we put the first nine trials of each session into the training set and the remaining six trials into the test set to train the model for each subject. For the subject-independent experiments, we employ leave-one-subject-out cross-validation. On SEED, we adopt two different experimental setups to segment the trials into samples. The first setup uses 1-s non-overlapping samples. For this setup, the learned local feature vector is used for emotion classification directly without the temporal modeling with Transformer, therefore the corresponding model is denoted as “DSSA Net w/o Transformer.” The second setup segments the trails into 4-s non-overlapping samples. For this setup, the local feature vectors are first learned from 1-s segments, then input into the Transformer for temporal modeling, the whole framework is denoted as “DSSA Net.”

**SEED-IV** contains four different categories of emotions, including happy, sad, fear, and neutral. The record consists of 15 participants. Three groups of experiments were designed for each participant on different days, each experiment contained 24 trails with six trails for each emotion category. EEG signals were recorded using the ESI NeuroScan System with 62 channels. For the subject-dependent experiments, we use the first 16 trials for training and the remaining eight trials for testing, ensuring coverage of all emotions (two trials per emotion category). The subject-independent experiments also adopt the leave-one-subject-out cross-validation strategy. These trials are segmented into 4-s non-overlapping samples, and DSSA Net first learns the local features from 1-s segments which are then input into the Transformer for temporal modeling.

**DEAP** is a open-source dataset containing multiple physiological signals with emotional evaluations for emotion recognition. It recorded the 40-channel EEG, Electrocardiogram (ECG), Electromyogram (EMG), and other bio-electrical signals of 32 subjects induced by watching 40 one-minute music videos of different emotional tendencies. The subjects then evaluated the videos' emotion category on the scale of 1–9 in dimension of arousal, valence, liking, dominance, and familiarity. In our experiments, we employ a 4-s non-overlapping sliding window, resulting in 15 EEG samples for each video. Valence and arousal are chosen as the criteria for emotional evaluation. To classify the samples into two categories (high/low) of arousal or valence, we set the threshold at a rating of five. 10-fold cross-validation are performed by dividing the samples of each participant into 10 equal parts. In each iteration, nine parts (32 × 9 × 4 × 15 = 17, 280 samples) are used for training the model and 1 part (32 × 4 × 15 = 1, 920) samples for testing. DSSA Net is adopted for local feature learning and temporal modeling.

We train our model on an NVIDIA TITAN RTX GPU. The cross entropy is adopted as the loss function. The Adam optimizer is used to minimize the loss function, the learning rate is set to 0.001. The architecture of the VA and HA modules consists of 2 CNN layers followed by a fully connected layer. The SA module comprises 1 CNN layer and a fully connected layer. The TA module consists of 2 Transformer encoder layers. Accuracy is used as the performance metric in our experiments. It is defined as the ratio of correctly predicted instances (both true positives and true negatives) to the total number of instances.

### 4.2 Compared models

To evaluate the effectiveness of the proposed model, we conducted comparisons with several advanced competitive models known for their efficacy in EEG-based emotion recognition:

- SVM (Suykens and Vandewalle, 1999): A Least Squares Support Vector Machine classifier.
- DBN (Zheng and Lu, 2015): Deep Belief Networks, which delve into critical frequency bands and channels.
- DGCNN (Song et al., 2018): Dynamical Graph Convolutional Neural Networks, adept at modeling multichannel EEG features.
- BiDANN (Li et al., 2018): Bi-hemispheres Domain Adversarial Neural Network, capable of mapping EEG feature data from both left and right hemispheres into discriminative feature spaces separately.
- RGNN (Zhong et al., 2020): Regularized Graph Neural Network, designed to exploit the biological topology among different brain regions and capture both local and global relations among EEG channels.
- IAG (Song et al., 2020): Instance-adaptive graph (IAG) method that flexibly constructs graph connections and utilizes multi-level and multi-graph convolutional operations to capture dynamic relationships among different EEG regions.
- 4D-aNN (Xiao et al., 2022): Four-Dimensional Attention-based Neural Network, proficient in fusing information from different domains and capturing discriminative patterns in EEG signals based on the 4D spatial-spectral-temporal representation.
- EESCN (Xu F. et al., 2024): A novel approach based on spiking neural networks for emotion recognition.
- EEG-GCN Gao et al. (2022): Spatio-temporal and self-adaptive graph convolutional network designed for EEG-based emotion recognition.
- ACRNN (Tao et al., 2020): Attention-based convolutional recurrent neural network to explore critical features for EEG emotion recognition.
- SOGPCN (Zhu et al., 2024): Self-organized graph pesudo-3D convolution based on attention and spatiotemporal convolution.
- 3D-CNN and PST-Attention (Liu et al., 2021): 3D-CNN within Positional, Spectral, and Temporal Attention modules.

In the following tables of experimental results, all the values of these works are taken from the referred publications.

### 4.3 Experimental results

The subject-dependent and subject-independent classification results on SEED and SEED-IV are shown in Table 1, where the upper part lists the results of the models without temporal modeling, while the lower part with temporal modeling. From the results without temporal modeling, one can see that the advanced models like DGCNN, RGNN, and IAG significantly improve the performance over the traditional machine learning models like SVM and DBN. However, they mainly consider the spatial information of EEG signals collected from different channels. DSSA Net performs the best among the compared models. This can be attributed to the following advantages of our proposed model. Firstly, it effectively integrates spatial and spectral features of the EEG signals. Moreover, the attention mechanism incorporated in the model allows it to focus on the most relevant channels and spectral bands of the EEG signal, which are salient on different emotions.

**Table 1:** Subject-dependent and subject-independent classification accuracy (mean/std) on SEED and SEED-IV (%), including results with DSSA Net with using Leaky ReLU activation function DSSA Net (lr).

**Category**	**Model**	**SEED**		**SEED-IV**	
		**Dependent**	**Independent**	**Dependent**	**Independent**
W/o temporal modeling	SVM (Suykens and Vandewalle, 1999)	83.99/9.72	56.73/16.29	56.61/20.05	37.99/12.52
	DBN (Zheng and Lu, 2015)	86.08/8.34	-	-	-
	DGCNN (Song et al., 2018)	90.40/8.49	79.95/9.02	69.88/16.29	52.82/9.23
	BiDANN (Li et al., 2018)	92.38/7.04	83.28/9.60	70.29/12.63	65.59/10.39
	RGNN (Zhong et al., 2020)	94.24/5.95	85.30/6.72	79.37/10.54	73.84/8.02
	IAG (Song et al., 2020)	95.44/5.48	86.30/6.91	-	-
	DSSA Net (lr) w/o transformer	96.18/5.36	85.89/6.30	-	-
	DSSA Net w/o transformer	96.32/5.31	85.97/6.26	-	-
W/temporal modeling	4D-aNN (DE) Xiao et al. (2022)	95.39/**3.05**	-	-	-
	EESCN (Xu F. et al., 2024)	-	-	79.65/**8.22**	-
	EEG-GCN (Gao et al., 2022)	85.65/7.49	77.30/8.21	-	-
	SOGPCN (Zhu et al., 2024)	95.26/3.52	**94.22/3.42**	-	-
	3D-CNN & PST-Attention (Liu et al., 2021)	95.76/4.98	-	82.73/8.96	-
	DSSA Net (lr)	96.58/5.41	87.01/5.96	84.98/11.89	75.83/7.20
	DSSA Net	**96.61**/5.39	87.03/5.97	**85.07**/11.93	**75.86/7.18**

The bold values indicate the best performance within each category.

From the lower part of Table 1, we can see that the 4D-aNN (DE) model obtains 95.39% subject-dependent recognition accuracy by using four-dimensional attention-based networks to capture spatial-spectral-temporal features. EESCN gets 79.65% subject-dependent classification accuracy in SEED-IV by introducing a NeuroSpiking framework that extracts spatio-temporal features. Our previous work, 3D-CNN and PST-Attention, achieves 95.76% subject-dependent classification accuracy in SEED and SEED-IV by incorporating a multi-dimensional attention mechanism that combines spatial, temporal, and spectral attention. These results highlight that incorporating temporal modeling consistently improves the performance of emotion recognition models. The DSSA Net further advances this by introducing directional spatial attention mechanisms that separately capture the unique contributions of anterior-posterior and left-right brain regions. This design allows for a more refined analysis of spatial dependencies, providing a deeper understanding of how different brain regions interact during emotional processing. The DSSA Net, builds on 3D-CNN and PST-Attention by incorporating directional spatial attention mechanisms, achieves the highest accuracy in subject-dependent emotion recognition on both SEED and SEED-IV datasets. The findings indicate that integrating multi-dimensional features is crucial for capturing the complex nature of EEG signals. Additionally, the attention mechanisms significantly boost the model's ability to focus on the most relevant parts of the EEG signals. This targeted focus helps DSSA Net maintain high performance even when dealing with the complex variability present in EEG data, making it particularly robust for subject-independent tasks.

To further explore the impact of different activation functions, we also evaluated a version of the DSSA Net with Leaky ReLU (lr) as the activation function, as shown in Table 1. While the overall performance of the DSSA Net with Leaky ReLU (lr) remains close to that of the standard DSSA Net, there is a slight variation in subject-independent accuracy, suggesting that the choice of activation function can slightly influence the model's ability to generalize across subjects. Specifically, the DSSA Net (lr) achieves 96.58% accuracy in the subject-dependent setting on SEED and 87.01% in the subject-independent setting, which is marginally lower than the standard DSSA Net. This observation reinforces the robustness of our model while showing that ReLU remains a competitive choice for balancing model complexity and performance.

It is worth noting that we focused on subject-independent analysis using the SEED and SEED-IV datasets due to their standardized evaluation protocols, which enable consistent comparisons across studies. In contrast, the DEAP dataset does not offer a universally accepted partitioning scheme for subject-independent testing, making such analysis less standardized and more challenging to compare fairly with existing work. Therefore, our initial study emphasized SEED and SEED-IV for subject-independent evaluations.

Table 2 shows the subject-dependent classification performances on the DEAP dataset for valence and arousal. Traditional methods like RF and SVM perform relatively poorly, with accuracies ranging from 63.09 to 69.65% for both valence and arousal. Deep learning methods such as CNN and GCNN show remarkable improvements, achieving accuracies around 77.64% for valence and 78.62% for arousal. More recent models like EEG-GCN and ACRNN demonstrate further enhancements. Particularly, the ACRNN model achieves accuracies of 93.72% for valence and 93.38% for arousal by using channel-wise attention and self attention. By leveraging a combination of vertical-horizontal-position attention and spectral-temporal mechanisms, our DSSA Net is capable of capturing intricate relationships between spatial and spectral features. This holistic approach results in a deeper integration of the different feature domains, which directly translates into improved emotion recognition accuracy. With the vertical-horizontal-position, spectral and temporal attention mechanism, our proposed DSSA Net achieves the highest accuracies for both valence and arousal, with mean accuracies of 94.97 and 94.73%, respectively. These results underscore the effectiveness of combining multiple attention mechanisms in a unified framework, making DSSA Net particularly adept at identifying the nuanced patterns in EEG signals that correspond to different emotional states.

**Table 2:** Subject-dependent classification accuracy (mean/std) on DEAP (%).

**Model**	**Valence mean/std**	**Arousal mean/std**
RF (Breiman, 2001)	63.93/5.08	66.37/10.29
SVM (Cortes and Vapnik, 1995)	63.09/6.22	69.65/13.41
DBN (Hinton et al., 2006)	73.67/7.54	78.04/6.18
CNN (LeCun et al., 1998)	74.52/6.09	78.08/6.23
GCNN (Kipf and Welling, 2016)	77.64/4.43	78.62/8.15
EEG-GCN (Gao et al., 2022)	81.77/5.58	81.95/7.71
ACRNN (Tao et al., 2020)	93.72/**3.21**	93.38/3.73
DSSA Net	**94.97**/4.23	**94.73 /3.27**

The bold values indicate the best performance within each category.

### 4.4 Ablation studies

To demonstrate the effectiveness of each module, we conduct ablation experiments on the SEED and SEED-IV datasets by comparing various configurations. The baseline method, which uses the flattened original DE feature without any attention module refinement, demonstrates the lowest performance among all configurations. This method serves as a non-attention baseline, providing a direct comparison to configurations with SA and PA modules. To further validate the significance of the differences in performance across various configurations, we conducted a paired-sample *t*-test between the results of the DSSA Net and each ablation configuration (SA, VA, HA, and PA). The *t*-tests were performed, using a significance threshold of *p* < 0.05. The analysis confirms that the integration of all three modules (HA, VA, and SA) in DSSA Net results in significant performance improvements compared to baseline. The results, as shown in Table 3, highlight the contributions of each module to the overall performance of the DSSA Net.

**Table 3:** The ablation experimental results (mean/std) of DSSA Net on SEED and SEED-IV (%).

**Schemes**	**Modules**			**SEED**		**SEED-IV**	
	**HA**	**VA**	**SA**	**Dependent**	**Independent**	**Dependent**	**Independent**
Baseline	X	X	X	86.37/6.71	77.57/7.21	68.21/12.67	65.39/9.29
SA	X	X	✓	92.92/6.67^*^	83.53/7.03^*^	75.73/11.89^*^	70.93/8.29^*^
VA	X	✓	X	92.27/7.36^*^	83.21/7.19^*^	75.46/12.31^*^	70.13/8.83^*^
HA	✓	X	X	93.29/8.11^*^	83.91/6.33^*^	76.03/12.31^*^	71.38/7.97^*^
PA	✓	✓	X	93.82/7.06^*^	83.16/6.73^*^	79.31/12.17^*^	72.06/9.03^*^
DSSA Net	✓	✓	✓	**96.61/5.39** ^*^	**87.03/5.97** ^*^	**85.07/11.93** ^*^	**75.86/7.18** ^*^

Differences are considered significant at *p* < 0.05. The bold values indicate the best performance within each category. Differences marked with ^*^indicate statistical significance at *p* < 0.05.

When only the SA module is added, we observe an improvement in performance, with accuracy rising from 86.37 to 92.92% on the SEED dataset and from 68.21 to 75.73% on SEED-IV, respectively for subject dependent emotion recognition. This comparison directly highlights the contribution of the spectral attention mechanism over non-attention methods, as the SA module better captures the relevant frequency band information for emotion recognition. For position attention, the inclusion of the VA module also leads to enhanced performance, indicating the importance of vertical integration for modeling brain regions. The HA module obtains even better results than the VA module, showing that the lateral dynamics of brain activity play an important role in emotion recognition. Furthermore, when the HA and VA modules are both adopted (referred to as PA module), the accuracies are overall increased. This suggests that attention to both left-right and anterior-posterior brain areas is crucial for accurate emotion recognition. The integration of directional attention modules (VA and HA) enables the DSSA Net to selectively emphasize features from specific brain regions, providing a distinct advantage over undirected models that do not differentiate between these spatial dimensions. As shown in Table 3, the addition of VA and HA modules significantly improves accuracy, demonstrating the benefit of leveraging directional information in the EEG-based emotion recognition task. Notably, when all the three modules (HA, VA, and SA) are integrated to form the complete DSSA Net, the highest accuracies across all tasks are achieved. These results emphasize that by incorporating spectral attention, the model gains significant improvements over the non-attention baseline, validating the value of weighting frequency band information for enhanced emotion recognition.

Table 4 shows the ablation results of DSSA Net on the DEAP dataset, evaluating the contributions of the Horizontal Attention (HA), Vertical Attention (VA), and Spectral Attention (SA) modules. The baseline model, which lacks any attention mechanisms, achieves the lowest performance with 78.68 and 78.22% accuracy for Valence and Arousal, respectively. Adding the SA module improves performance significantly, achieving 85.72% for Valence and 86.12% for Arousal, highlighting the importance of spectral information in emotion recognition. The VA and HA modules alone also improve accuracy, with HA slightly outperforming VA, suggesting that lateral dynamics in brain activity have a greater impact on emotion classification. The combination of HA and VA (PA module) further enhances the performance to 90.19% for Valence and 91.06% for Arousal, indicating that considering both anterior-posterior and left-right spatial differences is beneficial. The complete DSSA Net, which integrates HA, VA, and SA, achieves the best performance with 94.97% for Valence and 94.73% for Arousal, demonstrating the effectiveness of combining spectral and spatial attention for capturing complex emotional representations.

**Table 4:** The ablation experimental results (mean/std) of DSSA Net on DEAP (%).

**Schemes**	**Modules**			**DEAP**	
	**HA**	**VA**	**SA**	**Valence**	**Arousal**
Baseline	X	X	X	78.68/7.63	78.22/7.58
SA	X	X	✓	85.72/6.97^*^	86.12/6.33^*^
VA	X	✓	X	85.19/7.31^*^	85.98/6.39^*^
HA	✓	X	X	86.93/7.12^*^	86.86/6.97^*^
PA	✓	✓	X	90.19/6.17^*^	91.06/5.61^*^
DSSA Net	✓	✓	✓	**94.97**/4.23^*^	**94.73/3.27** ^*^

Differences are considered significant at *p* < 0.05. The bold values indicate the best performance within each category. Differences marked with ^*^indicate statistical significance at *p* < 0.05.

### 4.5 Analytic visualization

**Figure 5:**
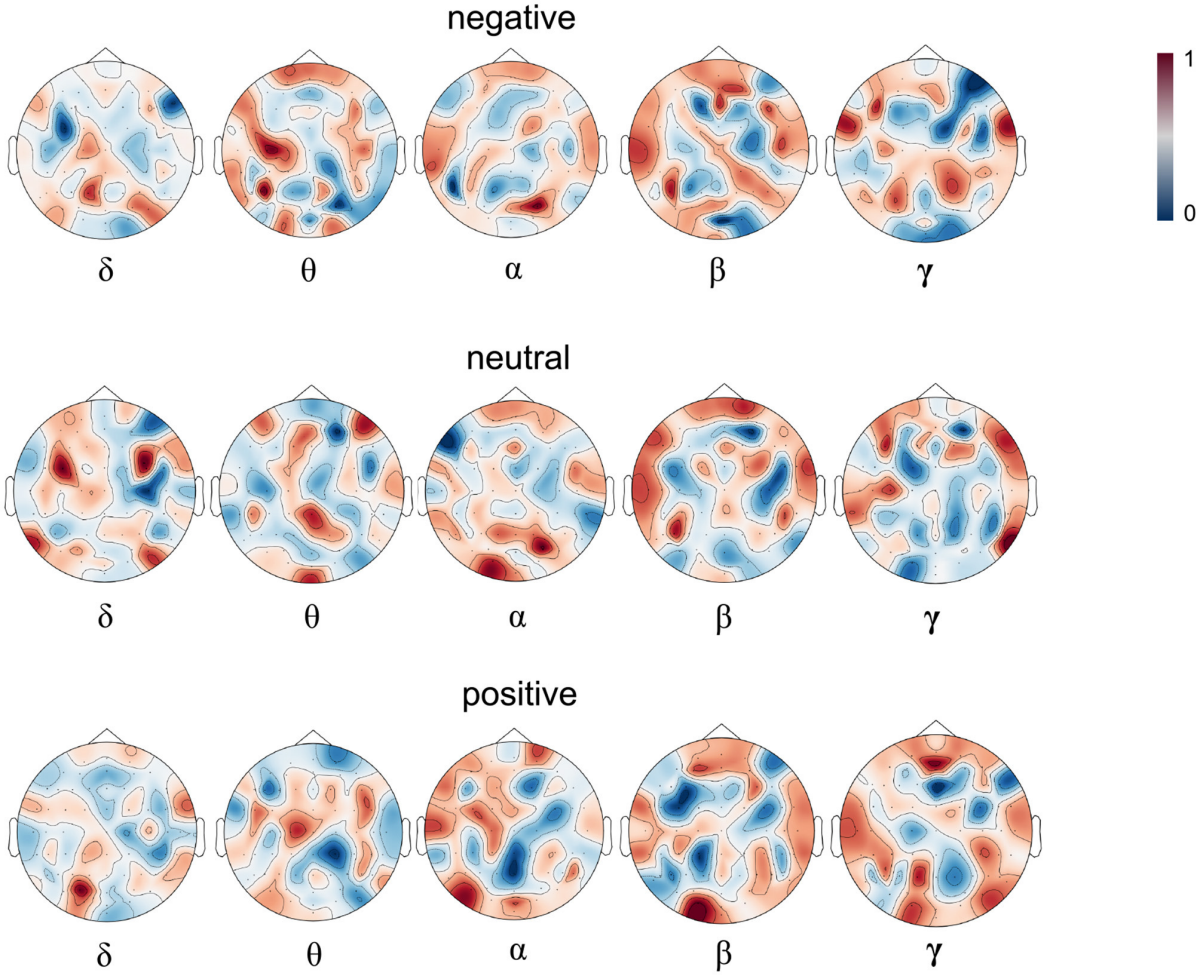
The attention heatmap *M* based on the samples of subject 1. The **upper, middle**, and **lower** parts correspond to negative, neutral and positive emotions and from left to right correspond to δ, θ, α, β, and γ bands respectively. Dark red denotes higher while dark blue denotes lower attention weight for the corresponding emotion.

In order to further interpret our experiment results, we visualize the attention heatmap *M* as shown in Figure 3. Figure 5 presents the averaged attention map for each emotion type over all the samples of the training set of the first subject in the SEED dataset. In the heatmap, red indicates high attention weights, suggesting higher activation levels in the corresponding brain regions; the deeper the color, the stronger the activation in that brain region. Blue indicates low attention weights, suggesting lower activation levels. From the heatmap, we can see that different emotional states correspond to distinct patterns of brain activation across the θ, α, β, and γ bands. For example, in the α band, which is typically associated with relaxation and calmness, higher activation is observed in the occipital and parietal lobes during calm emotional states. Conversely, the γ band, which is linked to high-level cognitive processing and positive emotional states, shows widespread activation across various brain regions when subjects experience positive emotions.

## 5 Conclusion

In this paper, we introduce a novel framework, the Directional Spatial and Spectral Attention Network (DSSA Net), which incorporates positional attention (PA), spectral attention (SA), and temporal attention (TA) modules to effectively extract and emphasize critical EEG features. The SA module emphasizes key spectral aspects by assigning weights to the differential energy spectrum of these bands, highlighting their importance. The PA module, which consists of vertical attention (VA) and horizontal attention (HA), targets brain regions in different directions that are activated by various emotions. Specifically, the VA branch is adept at detecting activations across the anterior-posterior brain regions, while the HA branch is to spotlight critical information from the left-right hemispheric areas. Moreover, a transformer encoder is employed to capture the dynamic evolution of emotional responses throughout different periods. Evaluation on three benchmark EEG datasets demonstrates that the DSSA Net surpasses most of the existing advanced methods in emotion recognition. In the future, we plan to leverage multimodal signals such as ECG and Galvanic Skin Response (GSR), alongside introducing networks like graph convolutional networks (GCNs) that are well-suited to the topological structure of EEG data, to enhance the recognition of emotional states by gaining deeper insights into brain dynamics.

## Data Availability

Publicly available datasets were analyzed in this study. This data can be found at: https://www.eecs.qmul.ac.uk/mmv/datasets/deap/; https://bcmi.sjtu.edu.cn/home/seed/.
